# Real Time 3D echocardiography (RT3D) for assessment of ventricular and vascular function in hypertensive and heart failure patients

**DOI:** 10.1186/1476-7120-10-27

**Published:** 2012-06-28

**Authors:** Maria Chiara Scali, Massimiliano Basso, Alfredo Gandolfo, Tonino Bombardini, Paolo Bellotti, Rosa Sicari

**Affiliations:** 1 Cardiologia Asl 2 Lucca, Lucca, Italy; 2 U.O. Cardiologia, Ospedale San Paolo, Savona, Italy; 3 CNR, Institute of Clinical Physiology, Pisa, Italy; 4 c/o Cardiology Unit, Ospedale San Francesco, Via dei Frati, 1, 55051, Barga, Lucca, Italy

**Keywords:** 3D Echocardiography, Ventricular Elastance.

## Abstract

**Background:**

Cardiac and systemic hemodynamics have been historically in the domain of invasive cardiology, but recent advances in real-time 3-Dimensional echocardiography (RT3D echo) provide a reliable measurement of ventricular volumes, allowing to measure a set of hemodynamic parameters previously difficult or impossible to obtain with standard 2D echo.

**Aim:**

To assess the feasibility of a comprehensive hemodynamic study with RT-3D echo.

**Methods:**

We enrolled 136 patients referred for routine echocardiography: 44 normal (N), 57 hypertensive (HYP), and 35 systolic heart failure patients (HF). All patients underwent standard 2D echo examination followed by RT3D echo examination, including measurement of left ventricular (LV) end-diastolic and end-systolic volumes and derived assessment of LV elastance (an index of LV contractility), arterial elastance (characterizing the distal impedance of the arterial system downstream of the aortic valve); ventricular-arterial coupling (a central determinant of net cardiovascular performance); systemic vascular resistances. Blood pressure was derived from cuff sphygmomanometer and heart rate from ECG.

**Results:**

A complete 2D echo was performed in all 136 patients. 3D echo examination was obtained in 130 patients (feasibility = 95 %). Standard 2D echo examination was completed in 14.8 ± 2.2 min. Acquisition of 3D images required an average time of 5 ± 0.9 min (range 3.5-7.5 min) and image analysis was completed in 10.1 ± 2.8 min (range 6–12 min) per patient. Compared to N and HYP, HF patients showed reduced LV elastance (1.7 ± 1.5 mmHg mL^-1^ m^-2^, p <0.001 vs N = 3.8 ± 1.3 and HYP = 3.8 ± 1.3) and ventricular-arterial coupling (0.6 ± 0.5, p < 0.01 vs N = 1.4 ± 0.4 and HYP = 1.2 ± 0.4). Systemic vascular resistances were highest in HYP (2736 ± 720, p < .01 vs N = 1980 ± 432 and vs HF = 1855 ± 636 dyne*s/cm^5^). The LV elastance was related to EF (r = 0.73, p < 0.01) and arterial pressure was moderately related to vascular elastance (r = 0.54, p < 0.01). The ventricular-arterial coupling was unrelated to systemic vascular resistances (r = −0.04, p NS).

**Conclusion:**

RT-3D echo allows a non invasive, comprehensive assessment of cardiac and systemic hemodynamics, offering insight access to key variables – such as increased systemic vascular resistances in hypertensives and reduced ventricular-arterial coupling in heart failure patients.

## Introduction

LV function, ventricular volumes, and ejection fraction are routinely assessed by standard 2D echo for early detection of cardiac disease, to monitor disease progression and to assess response to treatment. Standard echocardiographic evaluation of hemodynamic parameters shows an acceptable correlation with invasive measurements in population studies. However, in the individual patient, the dispersion of values may be so wide to limit clinical applications, mostly due to intra- and inter-observer variability of volumes measurements [1]. RT-3D improves accuracy of non-invasive evaluation of cardiac volumes limiting data scatter and provides reliable clinical guidance [2]. Moreover RT-3D echo, by accurate assessment of stroke volume (SV), allows to derive a set of hemodynamic measures usually difficult or impossible to obtain with 2D echo, such as LV elastance, arterial elastance, ventricular-arterial coupling and systemic vascular resistances [3-5].

The underlying idea of the present study was to take advantage of the superiority of 3D over 2D echocardiography in assessing LV volumes to derive more accurate non invasive estimates of cardiac-vascular function [6,7].

The purpose of this study was to evaluate the feasibility and time cost of RT-3D echo, as compared to standard 2D echo, in the setting of a primary care echocardiography laboratory.

We also evaluated RT3D derived cardiac and vascular hemodynamics in hypertensive and heart failure patients. In fact, RT3D-derived parameters such as left ventricular elastance or systemic vascular resistances reflect the complex interactions between the heart and its internal and external loads and are of emerging importance in the assessment and management of hypertension and heart failure [8].

### Study population

The study has been conducted in 3 different primary care cardiology outpatients clinic (Echocardiography Laboratories of Savona, of Lucca, and of Barga cardiology services). All exams were performed by the same cardiologist-echocardiographer who performed both 2D and 3D echo examinations. To minimize variability the same observed, acquired and analyzed all studies. The observer had undergone a dedicated 9 month training on 3-D and the variability observed in a consecutive set of 10 studies was consistently < 10 % for LV volumes. We initially considered 400 patients, referred for clinically driven Echo evaluation between May 2009 and June 2011. Sixty patients denied the consent to enter the 3D part of the study, 50 had technically difficult 2D-Echo examination; 154 had exclusion criteria conditions (such as previous myocardial infarction, valvular heart disease or age below 40 y). One hundred and thirty six patients were eventually included in the study. They complied with the inclusion criteria:

1. Sinus Rhythm;

2. Willingness to enter the study;

3. Technically good 2D echo study;

4. Clinical-echocardiographic diagnosis of no structural heart disease (N, with SBP ≤ 139 mmHg, diastolic blood pressure ≤ 85 mmHg, and a BMI ≤ 30 Kg/m2), free from major coronary risk factors, including diabetes, hypercholesterolemia, and cigarette smoking;

5. Clinical-echocardiographic diagnosis of essential hypertension (HYP), previously made according to standard criteria [9]: history of long standing high blood pressure, under active treatment with ACE-inhibitors (68 %), and/or diuretics (84 %), and/or ARBs (36 %), and/or Ca-channel blockers (25 %) with EF > 50 %;

6. Clinical-echocardiographic diagnosis of heart failure (HF): history of dyspnea on effort, under active treatment with ACE-inhibitors (86 %), and/or diuretics (78 %), and/or ARBs (28 %), and/or β-blockers (68 %), with EF < 40 %.

### Study protocol

Following standard 2D echo examination, patients underwent RT-3D echo with measurement of raw data of LV EDV and ESV and derived assessment of [10]: LV elastance (an index of LV contractility); arterial elastance (AE) (characterizing the distal impedance of the arterial system downstream of the aortic valve); ventricular-arterial coupling (a central determinant of net cardiovascular performance); systemic vascular resistances (SVR). Blood pressure was derived from cuff sphygmomanometer and heart rate from 1- lead ECG (on echo monitor). The medical records of all included patients were reviewed in detail by one investigator to identify N, HYP and HF patients. For all patients, age, cuff blood pressure, height, weight, body mass index (BMI) and body surface area (BSA) were calculated and recorded.

Mean arterial pressure (MAP) was calculated as diastolic blood pressure + (systolic blood pressure - diastolic blood pressure/3). Mitral regurgitation and pulmonary arterial pressure were estimated from standard 2D echo. Forty four patients with no overt cardiac disease, a SBP ≤ 139 mmHg, and a BMI ≤ 30 Kg/m2, constituted the N group. Fifty seven subjects with hypertension but no HF constituted the HTN group. Thirty five patients with a clinical diagnosis of heart failure and EF below 40 % constituted the systolic HF group.

## Methods

### Two-dimensional echocardiography

Standard 2-dimensional echocardiography was performed according to the recommendation of the European Association of Echocardiography [5] using a Philips I33 scanner equipped with a phased array S5-1 1.3-3.6 MHz probe with second harmonic capability. Left atrial dimensions (parasternal and 4 chambers view), LV end-diastolic volumes (EDV) and end-systolic volumes (ESV) were measured. Ejection fraction (EF), stroke volume (SV), and cardiac output (CO) were calculated according to standard formula [5]. The echocardiogram was considered adequate if ≥ 13 of the maximum 16 segments were visualized in at least 1 projection.

### Three-dimensional echocardiography

Real time 3-dimensional echocardiography images were recorded with a Philips I33 equipped with a X3-1 1‐3 MHz matrix–phased array transducer in a 60 × 70 pyramid shaped volume containing the entire left ventricle. Volumetric data were obtained only from the apical window and displayed as conventional 2D apical which were digitalized with final interpretation made off –line with manual identification of chambers contours in selected image. Four cardiac cycles were stitched together to obtain LV volumes.

3D-images were obtained soon after completing the 2D study using the same echocardiographic machines with a fast switch between the 2 probes [4].

BP and HR were taken simultaneously during volume assessment, from cuff sphygmomanometer and from EKG, respectively.

### Data acquisition

LV EDV and ESV were measured from apical four- and two-chamber view, using the biplane Simpson-method [5]. Only representative cycles with optimal endocardial visualization were measured and the average of three measurements was taken. The endocardial border was traced, excluding the papillary muscles. The frame captured at the R wave of the ECG was considered to be the end-diastolic frame, and the frame with the smallest left ventricular cavity the end systolic frame.

### Arterial elastance and ventricular-arterial coupling

Ventricular arterial coupling was derived by the ratio of LV systolic elastance index (systolic pressure/end-systolic volume index) to arterial elastance (ratio of end-systolic pressure by stroke volume). Effective arterial elastance (EaE), characterizing the distal impedance of the arterial system downstream of the aortic valve, was estimated as end-systolic pressure (ESP) divided by stroke volume (SV) and expressed as SP/ESV index = mmHg/mL/m^2^. ESP was estimated as systolic pressure times 0.9 [7]. Because stroke volume (and input impedance) varies directly with body size, arterial elastance was corrected for BSA (EaE) to better reflect differences in arterial properties with age and between the genders adjusted for differences in body size [11]. Of note ventricular-arterial coupling is ventricular elastance/arterial elastance, which can further be described as: ESP/ESV divided by ESP/SV: the pressure terms in the numerator and the denominator cancel out, and ventricular-arterial coupling equals to stroke volume/end-systolic volume.

### Systemic Vascular Resistance (SVR)

SVR were calculated according to the traditional formula:

(1) $$\text{SVR}=80*\left(\text{MAP}-5\right)/\text{CO}\text{,}$$

where 5 is an approximation of the right atrial pressure and MAP is mean arterial pressure and CO is Cardiac Output. Vascular resistance is a term used to define the resistance to flow that must be overcome to push blood through the circulatory system. The resistance offered by the peripheral circulation is known as the systemic vascular resistance. Vasoconstriction (i.e., decrease in blood vessel diameter) increases systemic vascular resistance, whereas vasodilatation (increase in diameter) decreases systemic vascular resistance. Units for measuring vascular resistance are dyne*s/cm^5^.

### Systemic arterial compliance

Systemic arterial compliance was calculated as SV index/systemic arterial pulse pressure; where pulse pressure = SBP - DBP [10,12].

### Statistics

Software (SPSS 11 for Windows, SPSS, Chicago, Ill) was used for statistical analysis. The statistical analyses included descriptive statistics (frequency and percentage of categorical variables and mean and standard deviation of continuous variables). One-way ANOVA was used to compare continuous variables between groups with intergroup comparisons by Newman-Keuls test. The agreement between continuous or discrete data was tested by the Bland-Altman method and by the concordance correlation coefficient comparing the mean differences between the two methods of measurements and 95 % limits of agreement as the mean difference. A p value of 0.05 was considered as statistically significant.

## Results

Patients demographic and hemodynamic parameters are presented in Table 1.

**Table 1:** Study population and hemodynamic parameters

**N.**	**N**	**HYP**	**HF**
	**44**	**57**	**35**
**Females**	**32**	**22**	**14**
BSA m^2^	1.7 ±0.2	1.9 ± 0.2	1.9 ± 0.2
LVEF%	62 ± 6	63 ± 9	35 ± 5 *
LVEDV (ml/m^2^)	51 ± 14	50 ± 13	83 ± 28 *
LVESV (ml/m^2^)	19 ± 5	20 ± 8	56 ± 29 *
SBP (mmHg)	127 ± 15	147 ± 14 *	126 ± 18
DBP (mmHg)	70 ± 9	83 ± 8*	78 ± 11

* = p < .01 vs other groups.

Standard 2D Echo was performed in 136 (68 females) patients. Diagnostic quality images with RT-3D echo were obtained in 130 patients (feasibility 95 %) that were distributed as follows: no structural cardiac disease (N, 44), hypertension without heart failure (HTN, 56), and systolic heart failure (EF < 40 %) (HF, 30). The commonest reason for incomplete RT3D was ventricular dimension exceeding the area covered by the probe.

**Figure 1:**
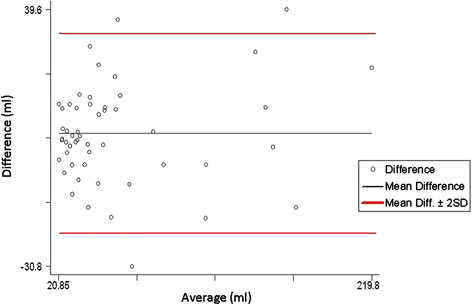
Bland-Altman plot for End-systolic volumes.

**Figure 2:**
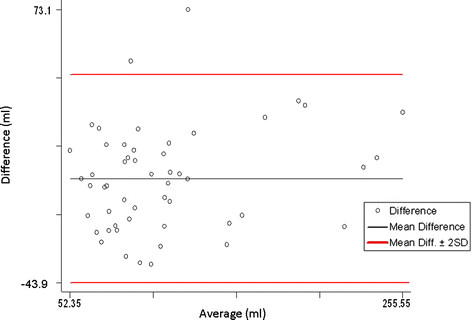
Bland-Altman plot for End-diastolic volumes.

LV volumes obtained with standard echo and with RT-3D echo were plotted according to the Bland-Altman method. End-systolic as well end-diastolic volumes showed a significant correlation (p < 0.001, Figure 1–2). In particular, a mean difference of 5.6 ml (95 % CI 1.8-9.4; r = 1.52; p = 0.28) for ESV and 6.1 ml (95 % CI 5.6-6.8; r = 0.21; p = 0.12) for EDV was found.

### Additional workload associated with RT3D echo

Standard 2D echo examination was completed in 14.8 ± 2.2 min. Acquisition of 3D images required an additional time of 5 ± 0.9 min (range 3.5 min/7.5 min) and image analysis was completed in 10 ± 2.8 min (range 6.6 min-12.8 min) per patient. Therefore the completion of a RT3D echo study from imaging to analysis requires a time interval comparable to a standard 2D examination.

### Non-invasive hemodynamic assessment

**Figure 3:**
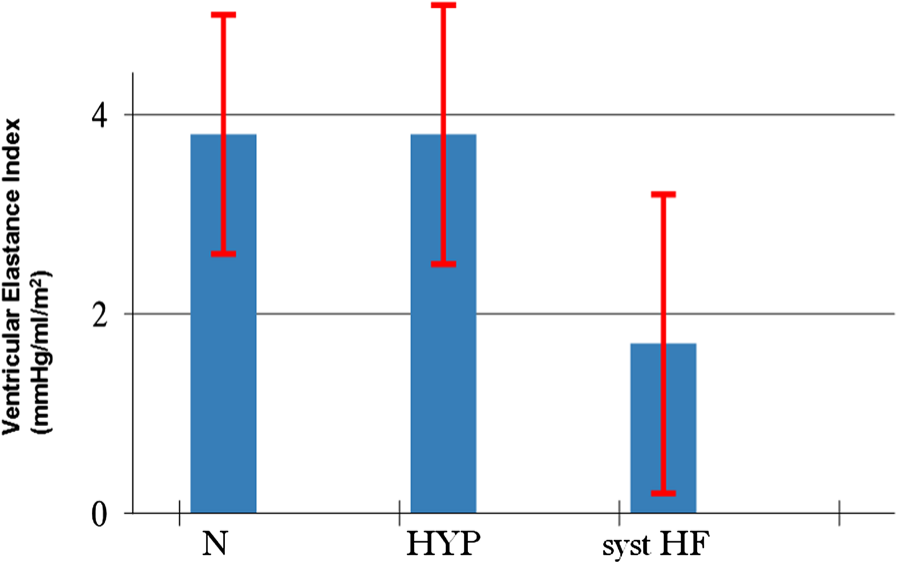
Ventricular elastance in the three study groups.

**Figure 4:**
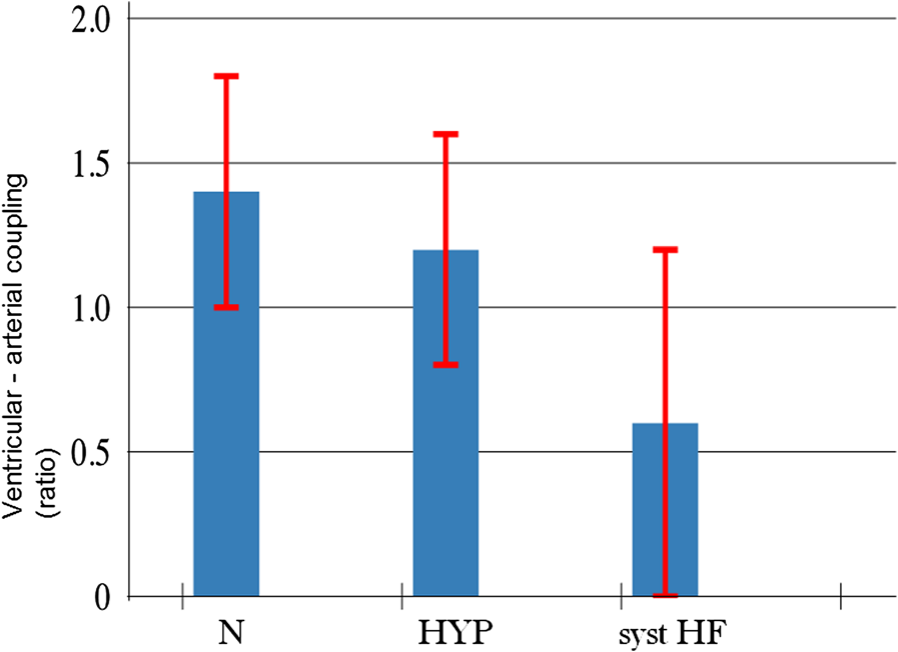
Ventricular-arterial coupling in the three study groups.

**Figure 5:**
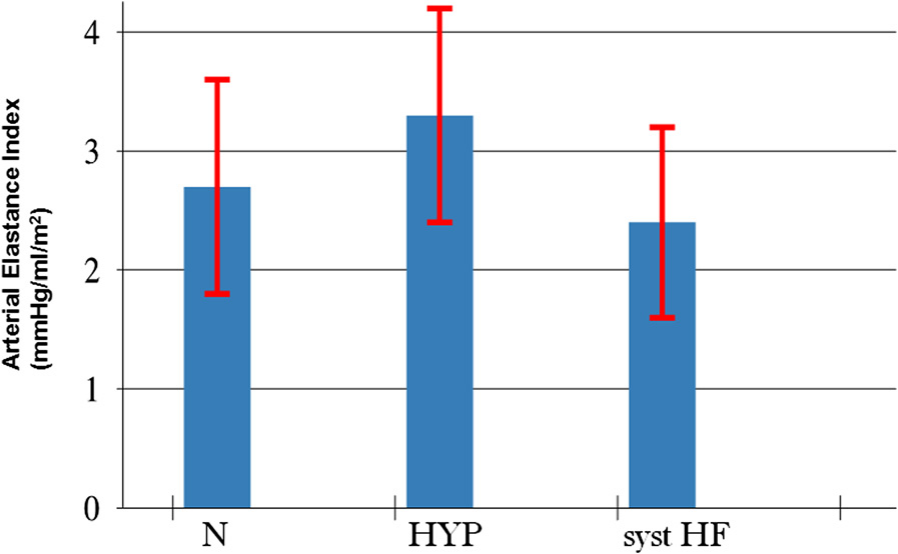
Arterial Elastance in the three study groups.

**Figure 6:**
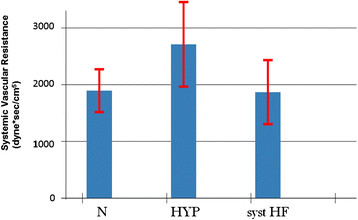
Systemic Vascular Resistances in the three study groups.

A reduced ventricular elastance was found in HF patients as compared to N and HYP (Table 2 and Figure 3). Ventricular-arterial coupling was also reduced in HF patients compared to N and HYP (Table 2 and Figure 4). Vascular elastance was significantly higher in HYP than in N and HF patients (Table 2 and Figure 5). HYP had the highest systemic vascular resistances (Table 2 and Figure 6)..

**Table 2:** Hemodynamic evaluation by RT3D echocardiography

	**N**	**HYP**	**Syst HF**
LV Elastance	3.8 ± 1.2	3.8 ± 1.3	1.7 ± 1.5 *
mmHg/ml/m^2^			
Arterial Elastance	2.7 ± 0.9	3.3 ± 0.9 *	2.4 ± 0.8
mmHg/ml/m^2^			
V-A Coupling	1.4 ± 0.4	1.2 ± 0.4	0.6 ± 0.5 *
SVR	1980 ± 432	2736 ± 720 *	1855 ± 636
dyne*s/cm^5^			

* = p < .01 vs other groups.

**Figure 7:**
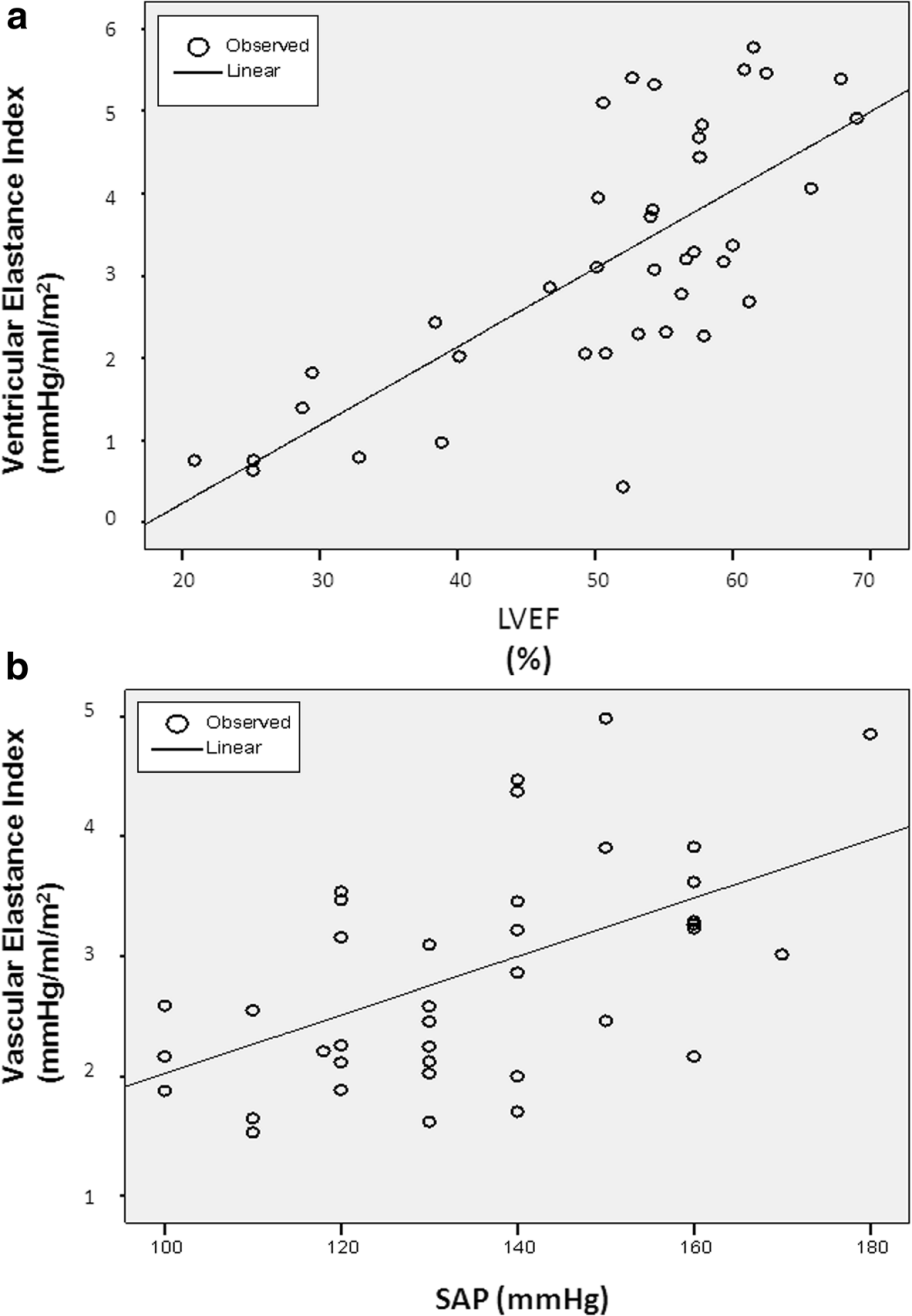
Ventricular elastance vs ejection fraction (Panel A) and Vascular elastance vs systolic pressure (Panel B).

A significant relation was found between LV elastance and EF (r = 0.73, p < 0.01) (Figure 7, panel A) and systemic arterial pressure was moderately related to vascular elastance (r = 0.54, p < 0.01) (Figure 7, panel B). No significant correlation was found between ventricular-arterial coupling and systemic vascular resistances (r = 0.04, p = NS) .

## Discussion

The primary purpose of this study was to test the feasibility of RT3D, and to estimate the time required to complete a RT-3D examination as compared to standard echo. The main finding of this study is that RT3D Echo, thanks to recent technological advances and to new, user-friendly software [10], can be performed in most patients referred for routine echo examination, and that RT-3D echo allows the assessment of cardiac hemodynamics in a variety of clinical conditions, in a relatively easy and quick fashion, at an acceptable time cost. Of interest, diagnostic quality RT-3D images were obtained in 95 % of patients with an acceptable acoustic window. The commonest reason for failed RT3D examination in this group was the presence of a markedly dilated left ventricle, exceeding the field of view of the 3D probe. Further technological advances promise to overcome this limitation.

Anticipated technological advances and new softwares will further shorten both acquisition time and analysis time, rendering RT-3D echo more and more attractive.

In keeping with previous studies, we found that LV elastance, vascular elastance, ventricular-arterial coupling, systemic vascular resistance, and other key hemodynamic parameters such as CO and SV, can be easily and quickly obtained with RT-3D echo in a number of cardiac conditions, including HYP, HF, and N patients [10-12].

3D echo makes it possible to capture the shape and function of the entire LV in a single data set. Compared with 2D echo, this is an advantage for LV quantification, since geometric assumptions of LV shape can be ignored. Moreover, 3D echo allows for manually aligning the displayed view to the true anatomical LV main axis, avoiding foreshortening and ensuing a precise identification of the LV apex.

In direct comparisons, RT-3D echo has been shown to be as accurate as contrast-enhanced 2D echo in left ventricular volume measurement. In addition, LV and RV volumes by 3D Echo have been reported to compare favorably with cardiac magnetic resonance (CMR) and Gated-SPECT imaging [13-16].

Compared to cardiac MRI, the currently accepted gold standard technique for LV quantification, 3D echo provides a good agreement for assessing EF with slightly underestimation of volumes, which may be attributed to the different modality in visualization of trabeculae and valves between the two techniques [17]

### Limitations

This study was not aimed to assess accuracy and reproducibility of 3D echo measurement of LV volumes, but to investigate the additional time costs of RT-3D images and its practicability .

Calculation of the end-systolic pressure/volume ratio would require the measurement of LV pressure in end-systole. Because only non-invasive measurements were available, systolic cuff pressure was used as a surrogate for end-systolic pressure. This certainly introduces an approximation, however, there is a tight relationship between peak and end-systolic pressure [18].

The conceptual novelty of the employed approach is limited, since already Bombardini et al. extensively showed that this approach can be usefully applied to non invasive ultrasound at rest and during stress such as exercise, dobutamine, dipyridamole and pacing [11,12,19]. However, we adopted firstly 3D echo which may introduce a crucial step-up in the feasibility and accuracy of the method, allowing to avoid inherent inaccuracies of the 2D-approach for volume calculations.

Hemodynamic variables assessed in this study may be affected by drug therapy. ß-blockers may reduce aortic wave reflection and improve left ventricular/vascular coupling, and 68 % of patients in the HF group were on ß-blockers [20]. However, this observation does not impact on our conclusions because we observed a reduced V/A coupling in the HF group as compared to N and Hyp patients, so, if anything, we are underestimating the difference.

## Conclusion

In this study we have shown that RT-3D can be performed in the vast majority of patients referred for echocardiographic examination at an acceptable time cost, comparable to standard 2D echo. Only patients with bad acoustic window or with markedly enlarged left ventricle may pose a difficult challenge. Reliable and detailed assessment of cardiac and systemic hemodynamics more than compensate for the time required for data acquisition and off-line analysis. Standard 2D echo will certainly remain the first line technique for LV assessment in the near future, given its large availability, its relative easiness, and the established role. However, when volumes are important and sequential testing is required, the 3D technique appears to be an attractive and practical alternative, offering also insight into variables of pathophysiological and potential clinical relevance, such as increased systemic vascular resistances in hypertensives and reduced left ventricular elastance in heart failure patients.

## Abbreviations

AE, Arterial elastance; ARBs, Angiotensin receptor blockers; BSA, Body surface area; EaE, Effective arterial elastance; EDV, End-diastolic volume; ESP, End-systolic pressure; ESV, End-systolic volume; HF, Heart failure; LV, Left ventricular; MAP, Mean arterial pressure; SVR, Systemic vascular resistance; SV, Stroke volume.

## Competing interest

The authors declare that they have no competing interests.

## Authors’ contributions

MCS, designed the study, collected and interpreted the data, carried out the statistical analysis. MB, AG and PB helped to collect the data. TB, was involved in designing of the study and revision of the manuscript critically for important intellectual content. RS, was involved in designing of the study and critical revision of the manuscript. All authors read and approved the final manuscript.
